# From dietary restriction to disease-targeted therapy: Sephience TM (sepiapterin) in phenylketonuria

**DOI:** 10.1097/MS9.0000000000004163

**Published:** 2025-10-17

**Authors:** Harshika Khaim Chandani, Sahira, Syeda Fadak Zahra Hujjat, Muhammad Saad Khan, Abubakr Mahmoud

**Affiliations:** a Department of Medicine, Jinnah Sindh Medical University, Karachi, Pakistan; b Liaquat College of Medicine and Dentistry, Karachi, Pakistan; c Allama Iqbal Medical College, Lahore, Pakistan; d Department of Medicine, University of Khartoum, Khartoum, Sudan

**Keywords:** phenylketonuria, precision medicine, rare metabolic disorders, Sephience, sepiapterin

## Abstract

Phenylketonuria (PKU) is a rare metabolic disorder caused by deficient phenylalanine hydroxylase, leading to toxic phenylalanine buildup and severe neurodevelopmental consequences if untreated. Despite advances in newborn screening and dietary management, treatment options remain limited and burdensome. The recent FDA approval of Sephience (sepiapterin) marks a major advancement in PKU care. Sepiapterin, a precursor of tetrahydrobiopterin (BH₄), enhances enzyme activity and stability, offering therapeutic benefit even in patients unresponsive to BH₄ alone. Results from the pivotal APHENITY Phase III trial demonstrated a substantial 63% mean reduction in blood phenylalanine and significant dietary liberalization, with 97% of participants able to increase natural protein intake safely. Importantly, 43% of prior non-responders to sapropterin showed clinical improvement, highlighting its potential to address key gaps in PKU management. With broad approval for children and adults, Sephience provides a disease-targeted therapy that extends beyond dietary restriction. While careful monitoring for adverse effects such as gastrointestinal symptoms and hypophenylalaninemia is required, this approval represents a transformative step in precision medicine. Sephience has the potential to redefine the standard of care and improve long-term quality of life for individuals living with PKU.

*To the Editor,*

Phenylketonuria (PKU) is a rare inherited metabolic disorder which is caused by a toxic buildup of phenylalanine due to a lack of phenylalanine hydroxylase^[1]^. And about 1 in 10 000 to 15 000 babies in the US are affected by PKU^[2]^. It can result in long-term problems, behavioral problems, and severe neurodevelopmental damage if untreated^[3]^. Although results have improved due to early diagnosis through neonatal screening and dietary management, treatment options are still scarce and difficult to maintain.

In this context, Sephience (sepiapterin), which was recently approved by the FDA, is a major breakthrough and presents a promising new treatment option for PKU patients that goes beyond dietary restrictions^[4]^. It is approved to treat children and adults with sepiapterin-responsive PKU, including hyperphenylalaninemia in patients as young as 1 month old^[4]^. Sepiapterin enhances both activity and stability of phenylalanine hydroxylase by supplying its cofactor BH₄ and acting as a pharmacological chaperone especially beneficial for PAH variants unresponsive to BH₄ alone. As a precursor of tetrahydrobiopterin (BH_4_), sepiapterin aims to improve phenylalanine metabolism by replenishing inadequate cofactor levels^[5]^.

This manuscript has been developed in accordance with the TITAN Guidelines (2025)^[6]^. The results from the pivotal APHENITY Phase III trial and its long-term extension revealed a remarkable mean reduction in blood phenylalanine of approximately 63%, and over 97% of trial participants could liberalize their diets with a 126% rise in natural protein intake while maintaining safe Phe levels^[7]^. Notably, about 43% of individuals who previously did not respond to Kuvan (sapropterin dihydrochloride) did benefit from sepiapterin, underscoring its potential to fill important gaps in current PKU therapies. Already approved across Europe since June 2025, Sephience received broad-label authorization covering all ages and disease severities, with rollout beginning in Germany, and additional regulatory reviews ongoing in Japan and Brazil. Store attention to its prescribing guidelines reveals detailed pediatric and adult dosing strategies, administration as an oral powder with food^[8]^.

In addition to expanding the therapeutic options, this advancement gives affected people hope for an improved long-term quality of life^[4]^.

The acceptance of Sephience emphasizes how crucial it is to keep up research, development, and funding for the treatment of uncommon diseases. Additionally, it emphasizes the necessity of greater awareness and accessibility to make sure that patients and their families can take full advantage of new treatment options.

With a therapy option that targets the disease’s fundamental pathophysiology, Sephience’s approval gives PKU patients new hope^[9]^. Despite the potential benefits of Sephience, patients should be closely watched for side effects such as headaches, diarrhea, stomach pain, and hypophenylalaninemia. Healthcare professionals should also be aware of possible drug interactions, including those involving PDE-5 inhibitors and levodopa^[10]^.

Although its demonstrated efficacy and broad labeling, Sephience has the potential to become a standard of care for PKU patients. The approval of Sephience represents a significant advancement in the treatment of PKU. It provides patients with a broader range of therapeutic options that go beyond strict dietary management, allowing for greater flexibility and improved daily living. This development underscores the progress in precision medicine and the dedication to meeting the long-term needs of individuals with PKU, ultimately ensuring better health outcomes and enhanced quality of life.

## Data Availability

No data were generated for this manuscript.
